# Arranged and non-arranged marriages have similar reproductive outcomes in Nepal

**DOI:** 10.1038/s41598-024-61467-8

**Published:** 2024-05-14

**Authors:** Elizabeth Agey

**Affiliations:** 1 Department of Anthropology, University of California, Santa Barbara, USA; 2 Department of Psychological and Brain Sciences, University of California, Santa Barbara, USA

**Keywords:** Human behaviour, Anthropology

## Abstract

Much of the evolutionary literature on mate choice presumes that individual mate preferences function to increase individual fitness, and this assumption has been confirmed in several experimental studies with animals. However, human mate choice, in many cultures, is heavily controlled by parents via arranged marriages, rather than the selection of the marrying individuals. Several studies have demonstrated that parents and offspring do not exhibit identical preferences for an in-law or spouse, respectively. If parental choice thwarts offspring’s evolved mate preferences from being expressed, then arranged marriages should reduce fitness. Using data from the Chitwan Valley Family Study, I examined whether having an arranged marriage, as compared to a non-arranged marriage, is associated with differences in total births, offspring survival to age 15, or interbirth intervals in Nepal, a culture with a rich history of arranged marriage. I find that there are no differences in any reproductive outcomes between arranged, co-selected, and self-selected marriages. These results indicate that individuals in arranged and non-arranged marriages may achieve similar fitness outcomes via different pathways, which may be unique to human mating systems.

## Introduction

In humans and other animals, preferences for particular mates, in contrast to random mating, presumably evolved because they increase reproductive fitness in their bearers. Despite the pervasiveness of studies on individual mate preferences, a large amount of human mate choice has occurred in a context where parents play an active role in selecting spouses for their children, often referred to as arranged marriage. Because parents and offspring have different fitness interests, and thus, different preferences in a spouse/in-law, individuals with mates selected by parents should theoretically experience differences in individual fitness compared to individuals with mates chosen individually. Thus, this study tests whether parental mate choice decreases reproductive success by comparing total births, offspring survival, and birth timing among women in arranged, co-selected, and self-selected marriages in a rural Nepali population.

Arranged marriage, is a widespread practice in which close relatives, usually parents, choose a marriage partner for their offspring^1,2^. Arranged marriage provides an opportunity to examine the effects of individual mate choice on fitness because the interests of parents and their offspring often do not fully align. Parents can increase their fitness via aggregate grandchild production, while their offspring gain larger fitness benefits through their own reproduction than through their siblings’^3^. When parents arrange a marriage they may focus more on family-level benefits of the match (e.g., social or financial resources), while their offspring should theoretically be more focused on traits that maximize their individual fitness (e.g., physical attraction). If parents and offspring express different preferences for fitness-relevant traits in an in-law or spouse, respectively, then arranged marriages and non-arranged marriages should have different fitness outcomes.

Copious ethnographic data suggest that parents and their offspring do not agree on preferred traits for an in-law or spouse. A comprehensive survey of the Human Relations Area Files database found that parents and offspring show disagreement over spouse choice in 85.4% of ethnographies analyzed, and this pattern was consistent in every world area^4^. Several studies, including in arranged-marriage cultures, show that people place more value on physical attractiveness in a spouse (a potential indicator of genetic quality or compatibility) than do their parents^5–10^. Conversely, parents in these studies seem to place more weight on similar cultural and religious backgrounds than do their children. Similar backgrounds support the building and strengthening of social ties, which would benefit the marrying offspring’s siblings and other family members and would thus be more important to parents than to each of their offspring individually^11^. Likewise, in a Chinese sample, parental matchmaking seemed to prioritize financial qualities of a spouse over mutual attraction between the couple^12^. Parent–offspring disagreement may also exist over marriage timing^13,14^, and offspring may desire later marriages to gain power over spouse selection or to avoid early reproduction. While there is also much overlap in fitness interests between parents and offspring, the traits that appear to produce disagreement are reproductively relevant. Thus, arranged marriage can be examined as an indicator of the fitness effects of constrained individual mate choice.

Despite ample cross-cultural evidence of parent–offspring disagreement over the qualities of a potential spouse, it is still unclear whether these disagreements have fitness consequences. A number of experimental (non-human) animal studies have attempted to test whether the opportunity to exercise mate preferences (versus assigning mates) increases reproductive success. These animal studies show several fitness benefits to free mate choice for both males and females, including better offspring survival^15–22^, faster reproduction^23–26^, improved offspring growth^27,28^, and resistance to infection^29^. Arranged marriage is not a perfect analogy to the experimental animal studies because spouse selection is not “random mating”—both arranged and non-arranged marriage involves partner choice, although by different parties. In spite of this, if human fitness is similarly tied to mate choice as in the experimental animal studies, then similar types of fitness outcomes should be affected when parents constrain offspring from exercising their preferences in arranged marriages.

However, previous work has not found clear support for this. For example, no differences in offspring survival between those in arranged and non-arranged marriages across or within three societies, the Yali, Tsimane, and Bhotiya^30^. Another study found that arranged couples in Indonesia had fewer live births and lower offspring survival, but there were no differences in other fitness measures indicative of offspring growth, such as birth weight, or parental investment, measured by breastfeeding duration^31^. Conversely, a study in China found that couples in parentally matched marriages had more children than individually-matched couples^32^.

These studies provide mixed evidence on whether humans show the predicted relationship between individual mate choice and higher reproductive success. This could simply be due to low-resolution measurement of individual input on spouse choice since these studies distinguish arranged and non-arranged marriages as dichotomous categories. In reality, parent and offspring choice does not always fall neatly into two discrete categories; for example, offspring may be asked to give final approval over their parents’ choice even in arranged marriages^1,33^. Thus, the expected fitness differences may only emerge when spouse choice is parsed into more categories better representing the range of parent and offspring influence. If humans do not show the predicted fitness differences, even with the inclusion of additional spouse choice categories, then humans may be utilizing alternative fitness strategies that mitigate the costs of constrained mate choice, and theories that human mate preferences function to increase individual fitness may need reexamination to account for the role of parents and others in the mate choice process.

While it is important to test the connection between mate choice and fitness for theoretical reasons, the connection between arranged marriages and reproductive outcomes is also important for public policy. Arranged marriages are sometimes assumed to have poor outcomes for women and children by NGOs and other international organizations. If arranged marriages have poorer offspring survival than self-chosen marriages after accounting for demographic differences between individuals, then these results could support those causes. Alternatively, if there are no differences in outcomes like offspring survival, then it might indicate that these practices are less harmful than assumed, and that resources could be allocated to alternative intervention programs.

This study compares total births, offspring survival, and interbirth intervals between women in arranged and non-arranged marriages to assess whether humans experience fitness benefits from choosing their own mates compared to parental mate choice. To examine these outcomes, I analyzed data from the Chitwan Valley Family Study of Nepal (CVFS)^34^. Because Nepal has been shifting from a tradition of arranged marriages to a system of non-arranged marriages^35^, these data include useful variation in marriage type in a large representative sample with rich demographic information, which can be used to control for confounding demographic differences between the types of people who end up in different types of marriages. Nepal also has a very low rate of divorce and remarriage^36^, making spouse choice an effectively permanent decision that is more likely to have detectable long-term fitness consequences. Using data from approximately 1,200 women in the CVFS, I examine three fitness outcomes that are improved by free mate choice in experimental animal studies or in previous studies testing the association between arranged marriage and fitness: total births, offspring survival, and interbirth intervals. If expression of individual mate preferences increases fitness, then I hypothesize that:

- (Analysis Set 1) women in arranged marriages will have fewer total births,
- (Analysis Set 2) women in arranged marriages will have lower offspring survival,
- (Analysis Set 3) women in arranged marriages will have longer interbirth intervals (i.e., a slower rate of reproduction).

Each of these outcomes will be examined with both dichotomous marriage categories (arranged/non-arranged) and trichotomous marriage categories (arranged/co-selected/self-selected) to investigate whether previous studies have returned mixed results simply due to categorization of marriages.

## Results

### Descriptive statistics

**Figure 1:**
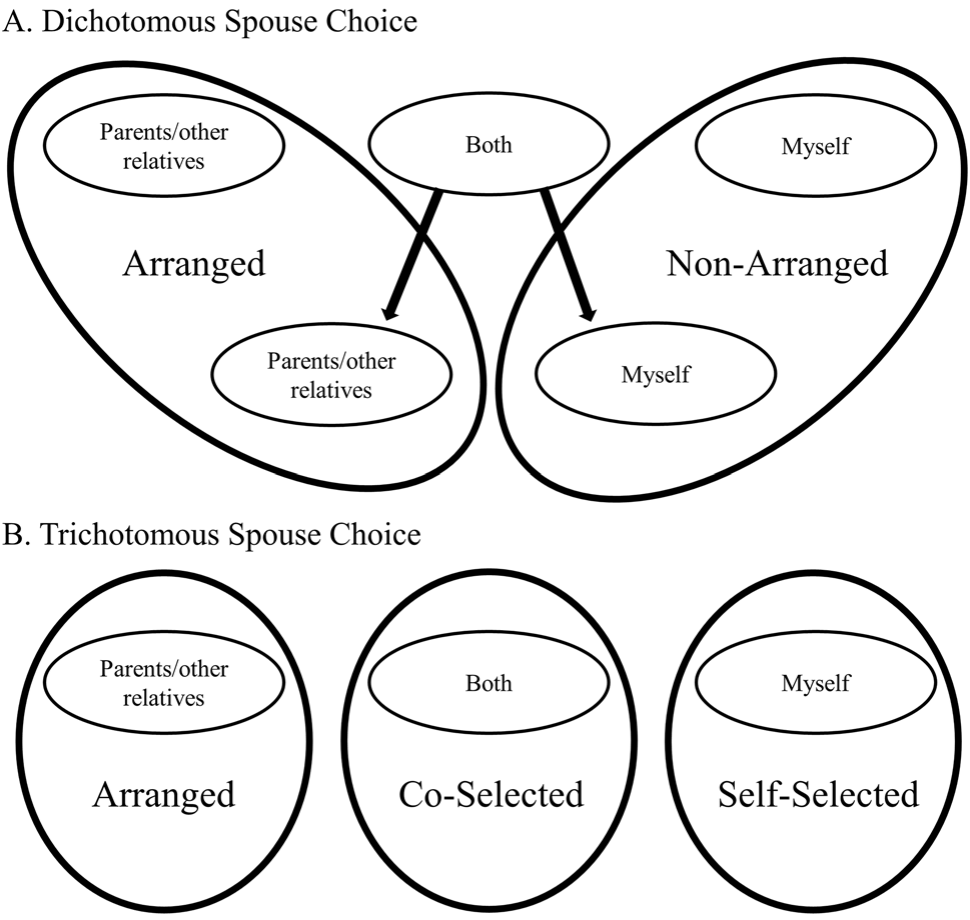
Marriage type coding schemes based on responses to the question “Who primarily was responsible for choosing your spouse?”. Respondents who answered “both” to the initial question were asked a follow-up question to identify who had more influence—themselves or parents. Responses to the follow-up question were only used to determine dichotomous marriage categories. Dichotomous choice is used in models 1a-b, 2a-b, and 3a-b and trichotomous choice is used in models 1c, 2c, and 3c-d.

Marriage category is based on answers to the question “Who primarily was responsible for choosing your spouse?”. Respondents who answered “Both [parents and myself]” were asked a follow-up question to determine whether their parents or themselves had more influence. The coding scheme for dichotomous and trichotomous marriage categories based on these responses is illustrated in Fig. 1 and is described in more detail in the Methods. Descriptive statistics for trichotomous marriage types are presented in Table 1. In total, 36.9% of marriages were arranged by parents or relatives in this sample, 39.6% of marriages were self-selected, and 23.5% were co-selected by parents and offspring. In the 1298 women included in analyses, 78.7% are over age 35 and 43.2% are over age 45, indicating that a substantial proportion of women in the analyses have completed reproduction. Respondents who chose their own spouse tended to be younger at the time of data collection (means: arranged = 47.3 years, co-selected = 39.0 years, and self-selected = 38.8 years). This likely indicates a secular change in marriage practices, with movement away from arranged marriages and toward non-arranged marriages. Those who co-selected a spouse with their parents had higher mean levels of education (means: arranged = 5.5 years, co-selected = 8.1 years, self-selected = 5.7 years). Women in arranged and co-selected marriages had slightly higher income levels (means: arranged = 3.3, co-selected = 3.4, self-selected = 3.1). Women in arranged marriages tended to marry at younger ages than those in non-arranged marriages (means: arranged = 20.1 years, co-selected = 22.5 years, self-selected = 21.6 years). Those in arranged marriages tended to have more children on average (means: arranged = 4.1 children, co-selected = 2.6 children, self-selected = 3.0 children). Women in arranged marriages used birth control for fewer years on average (means: arranged = 2.4, co-selected = 3.5, self-selected = 3.1). Of the respondents who said they jointly chose their spouse with their parents (i.e., co-selected spouses), 42.6% subsequently said their parents had more influence, while 41.5% said they had more influence themselves. Summary statistics for the dichotomous marriage type categories are available in Supplementary Table S1.

**Table 1:** Mean and standard deviation for demographic characteristics by marriage type for married women in the Chitwan Valley Family Study.

	Arranged (n = 479)		Co-Selected (n = 305)		Self-Selected (n = 514)	
	Mean	SD	Mean	SD	Mean	SD
Age	47.3^a^	9.6	39.0^b^	9.7	38.8^b^	10.8
Age at marriage	20.1^a^	4.7	22.5^b^	4.1	21.6^c^	3.9
Marriage year*	1982^a^	11.4	1993^b^	10.7	1991^b^	10.8
Age at first birth	23.9^a^	4.9	24.6^a^	4.0	23.9^b^	4.2
Total children	4.1^a^	2.3	2.6^b^	1.5	3.0^c^	2.1
Total surviving children	3.7^a^	1.9	2.4^b^	1.3	2.7^c^	1.8
Total years on birth control	2.4^a^	4.1	3.5^b^	4.4	3.1^b^	4.1
Years attended school	5.5^a^	4.4	8.1^b^	4.3	5.7^a^	4.4
Household income level**	3.3^a^	1.4	3.4^a^	1.4	3.1^b^	1.3

Differing superscripts indicate significant differences (p < 0.05) across each row. Shared superscripts indicate no significant differences between marriage types.

*Marriage years are converted to Gregorian calendar years by subtracting 56 from the Nepali calendar years used in the CVFS. Nepali calendar year means are 2038 (Arranged), 2049 (Co-Selected), and 2047 (Self-Selected).

**Ordinal categories ranging from 1 to 7, see “Methods” for the corresponding categories.

In addition to these demographic summary statistics, we also examined patterns of love and marital disagreement across marriage types. Women in arranged, co-selected, and self-selected marriages experienced similar levels of love and marital disagreements (Supplementary Tables S2 and S3).

### Analyses

Table 2 summarizes all following analyses.

**Table 2:** Summary of analyses.

Model	Outcome	Spouse choice coding	Model type	Control variables
1a	Number of births	Dichotomous	Poisson GLM	Age, Household Income, Education, Age at First Marriage
1b		Trichotomous		
2a	Offspring survival to age 15	Dichotomous	Discrete time survival analysis	Time, Mother’s Age at Birth Event, Child’s Birth Year, Mother’s Education, Household Income
2b		Trichotomous		
3a	First birth interval	Dichotomous	Discrete time survival analysis	Time, Time Squared, Age, Age at Marriage, Education, Household Income
3b	Second birth interval			
3c	First birth interval	Trichotomous		
3d	Second birth interval			

#### Analysis set 1

**Figure 2:**
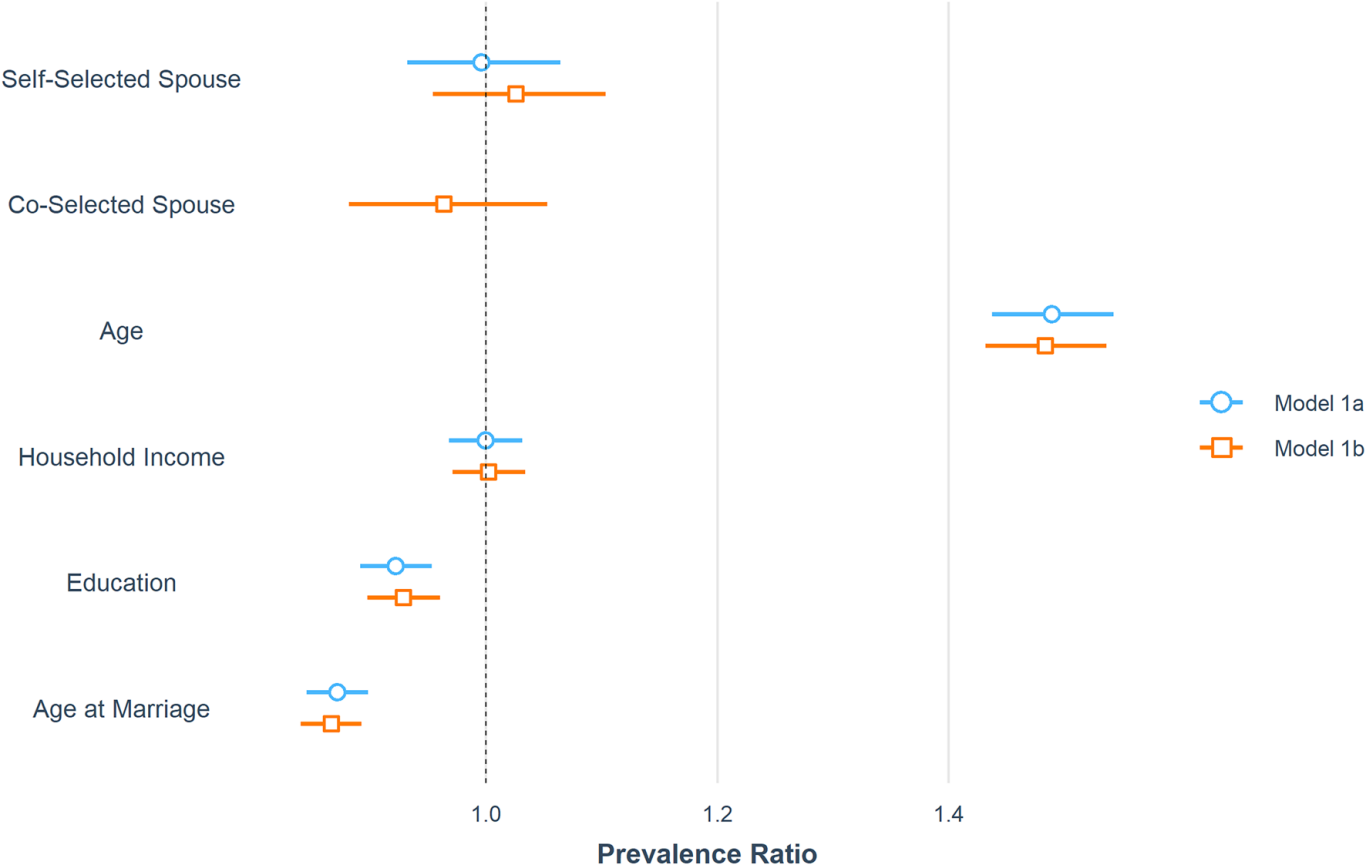
Plotted results of models 1a (dichotomous choice) and 1b (trichotomous choice), variables affecting number of births. Coefficients are converted to prevalence ratios and the bars represent 95% confidence intervals. Reference spouse choice group for all models is arranged marriage. For more detail, see Supplementary Table S4.

The results of models 1a and 1b are illustrated in Fig. 2 (for a table of results, see Supplementary Table S4). Type of marriage, whether arranged or non-arranged, had no effect on number of births. These results did not change when running the analyses with three marriage categories rather than two (Analysis 1b).

#### Analysis set 2

**Figure 3:**
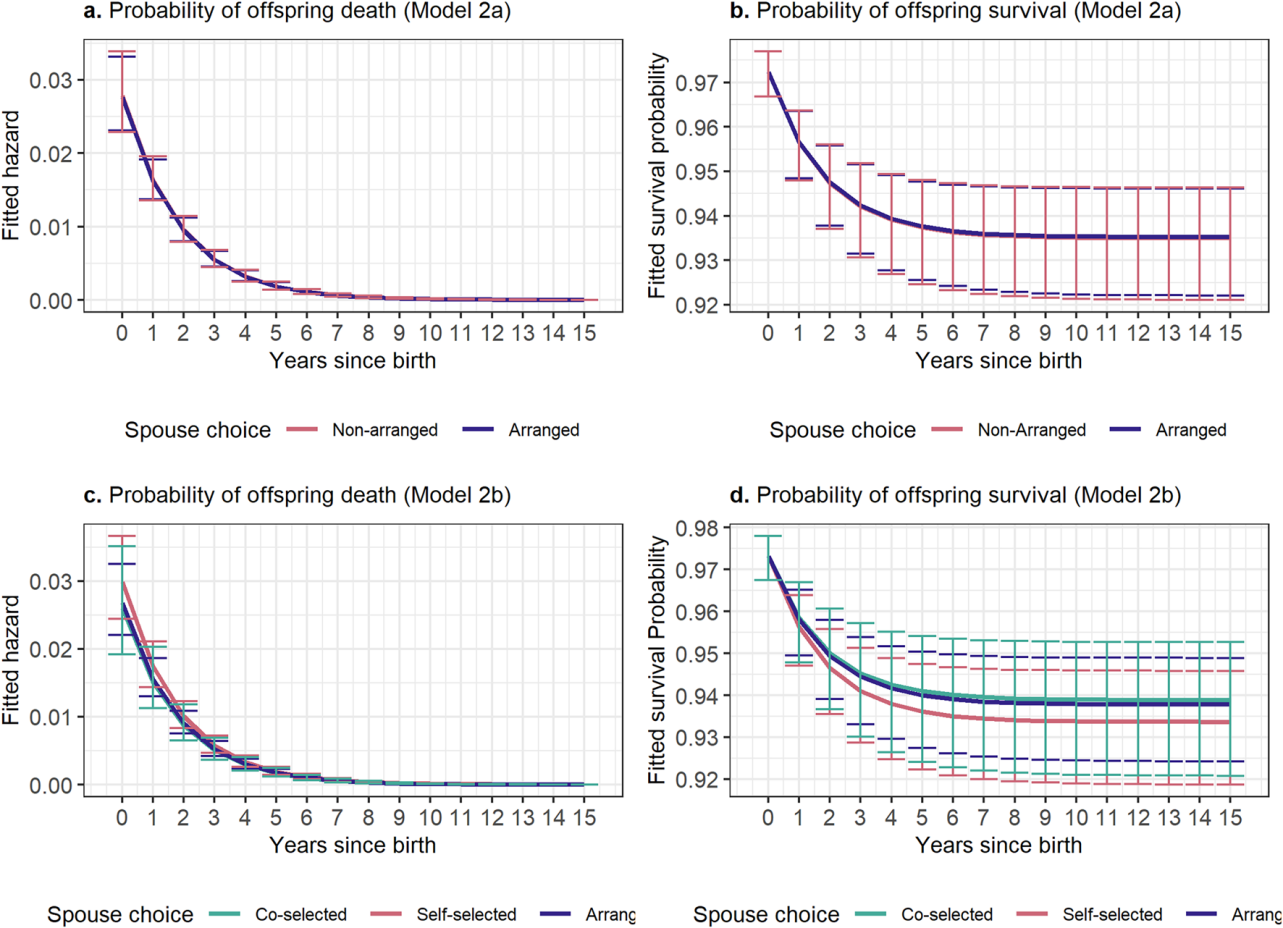
Fitted hazards and survival probabilities from Models 2a (dichotomous choice) and 2b (trichotomous choice), measuring offspring survival to age 15. Error bars represent 95% confidence intervals. All models are adjusted for mother’s age at the time of the birth, child’s birth year, mother’s education, and household income level.

Offspring survival to age 15, modeled using discrete-time survival analysis^37^, did not show any differences between marriage types in either the dichotomous or trichotomous choice. Figure 3 shows the predicted hazards (risk of offspring death) and survival curves (probability of surviving to each age) by marriage type. Supplementary Table S5 shows the model results from which these predictions were derived. Again, the dichotomous and trichotomous spouse choice models did not show any differences in results.

#### Analysis set 3

**Figure 4:**
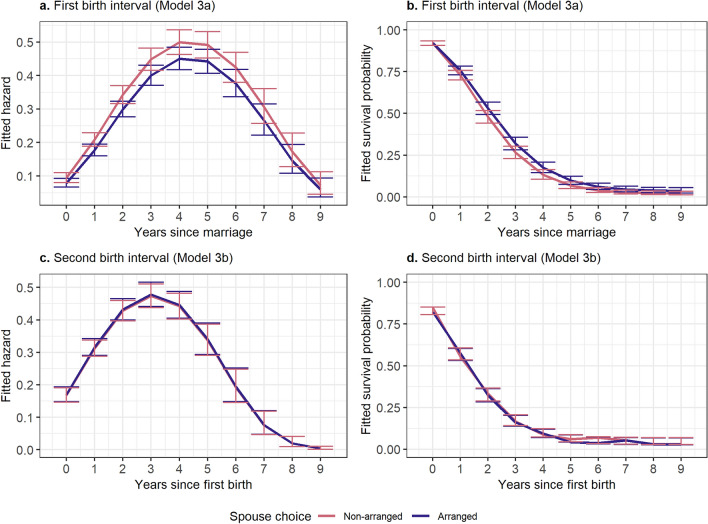
Fitted hazards and survival probabilities from Models 3a and 3b, measuring first and second birth interval, respectively, using two levels of spouse choice. Error bars represent 95% confidence intervals. All models adjusted for age, age at marriage, education, and household income.

**Figure 5:**
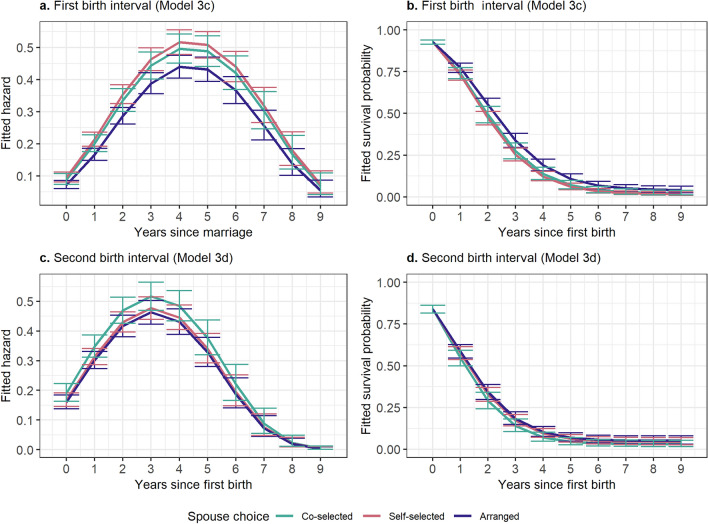
Fitted hazards and survival probabilities from Models 3c and 3d, measuring first and second birth intervals, respectively, using three levels of spouse choice. Error bars represent 95% confidence intervals. All models adjusted for age, age at marriage, education, and household income.

Interbirth intervals were also investigated using discrete time survival analyses to see if there were differences in timing of reproduction between those in arranged and non-arranged marriages. Model estimates are provided in Supplementary Table S6; these estimates were converted to predicted hazards and survival curves (represented in Figs. 4 and 5) to determine the relationships between marriage type and time. There are no significant differences in first birth interval or second birth interval between those in non-arranged and arranged marriages. Running this analysis with three marriage categories also did not show any significant differences in first birth interval or second birth interval by marriage type (Fig. 5).

### Parent–offspring disagreement in the CVFS

To help interpret the above results, I completed a post-hoc exploratory tabulation of the level of parent–offspring disagreement across the sampled communities, although it was not possible to assess parent–offspring disagreement for the sample of currently married women analyzed above. The CVFS asks a small set of questions that can be used to estimate the level of parent–offspring disagreement between unmarried men and women and their parents. Respondents were asked to choose the first and second most important traits in a spouse for themselves or for their child, respectively, from a list of four qualities: physical beauty, good education, high paying job, and someone you (your son/daughter) love(s). Based on a simple tally of these data, both parents and offspring overwhelmingly prefer good education (offspring = 52.2%, parents = 54.3%) and high paying jobs (offspring = 28.6%, parents = 29.3%) as the most important trait in a potential spouse/in-law, indicating some overall agreement (see Supplementary Table S7).

**Figure 6:**
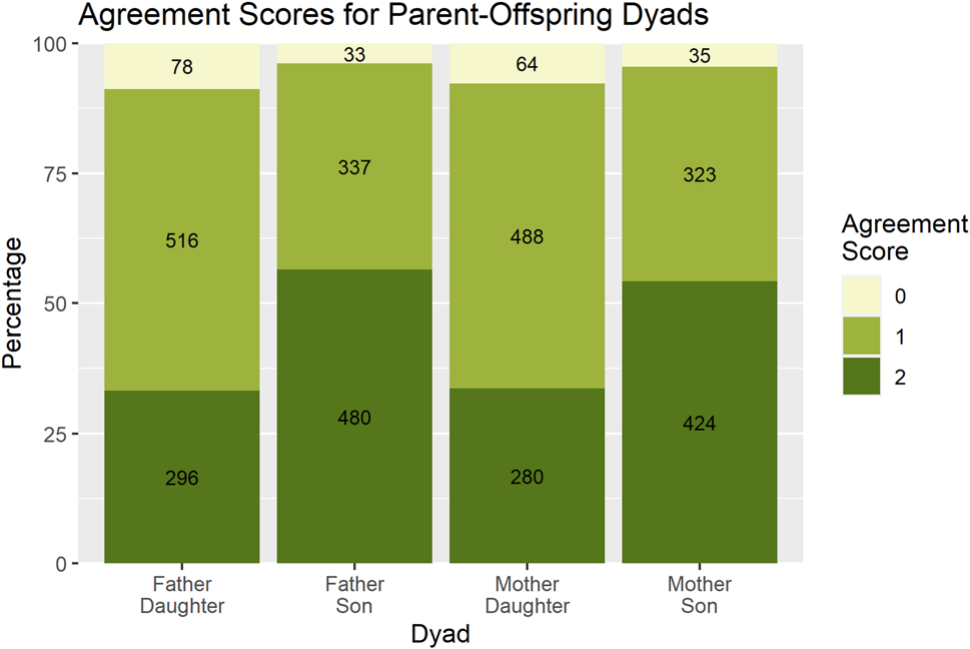
Agreement scores for each type of parent–offspring dyad. Bars represent percentages while the numbers in each bar represent counts of the dyads in each category. An agreement score of 2 indicates the dyad agrees on both of the most preferred traits (disregarding order) while an agreement score of 0 indicates the dyad did not agree on either of their most preferred traits.

To further investigate, I calculated an Agreement Index for 3,307 parent–offspring dyads, which counts whether a dyad agrees on 0, 1, or 2 of their top two preferred qualities (from the same list of qualities above). Sorting by sex (See Fig. 6), Mother-Son and Father-Son dyads agree on the top two qualities 54% of the time and 57% of the time, respectively. Mother-Daughter and Father-Daughter dyads agree on the top two traits 34% of the time and 33% of the time, respectively. Complete disagreement (i.e., an Agreement Index of 0) was uncommon across all types of dyads (Range: 3.9–8.8%), indicating that there is generally some amount of parent–offspring agreement. There was also hardly any disagreement over ideal marriage age within the 3307 parent–offspring dyads, with 52% of parents and offspring perfectly agreeing on marriage age and 80% of dyads differing in their opinions by less than one year of age (Supplemental Fig. S1). As with the agreement index, parent-daughter marriage age preferences diverged more than parent-son marriage age preferences. This might indicate that more disagreement could be produced when daughters are marrying than when sons are marrying. Thus, if parents have equal influence on daughters’ and sons’ marriages, daughters would be less likely to match their evolved mate preferences and should be more likely than men to show fitness differences based on marriage type.

## Discussion

Despite the larger sample size, an additional category of marriage type, and adjustment for potentially confounding socioeconomic variables compared to previous studies^30,31^, no differences were found between arranged- and non-arranged marriages in total births, offspring survival, or first or second interbirth intervals. These results are surprising because individuals prevented from expressing their own evolved mate preferences via parental mate choice should have different fitness outcomes than those who choose their own mates independently. There are many potential reasons for these results, discussed below.

### Measurement of marriage type

Each fitness outcome was examined with dichotomous and trichotomous marriage categories to explore whether previous research found no/conflicting effects of marriage type on fitness simply because dichotomous marriage categories did not capture the range of choice in actual marriages^1^. However, expanding the analyses to include three marriage types (Models 1b, 2b, 3c-d) still showed no differences across all fitness measures.

Still, the criteria used to form categories, rather than the number of marriage categories, may obscure differences. Parents who singlehandedly chose a spouse for the respondent and parents who chose a spouse but obtained approval from the respondent could have both been categorized as “parents had more influence” over spouse choice in this data set, despite differing levels of offspring influence. Similarly, some individuals whose parents chose their spouse but obtained offspring approval before marriage could have categorized their marriage as co-selected while others in the same scenario may have categorized their marriage as arranged. Further research designed to examine the fitness benefits of mate choice should aim to measure several facets of the spouse choice process to create a more meaningful measure of the degree of offspring mate choice.

Furthermore, it is possible that other factors related to spouse choice are more relevant to fitness outcomes. For example, feelings of love should be related to reproductive success^38^. Reported feelings of love toward a spouse were fairly consistent across marriage types in the CVFS sample (Supplementary Table S3). Likewise, frequency of disagreements was also similar across marriage types in the CVFS (Supplementary Table S4), indicating that compatibility may be independent of marriage type in this sample. To check, I also ran model 1b (trichotomous spouse choice) with an additional covariate for love and an interaction between marriage type and love to see whether feelings of love had an impact on total births, but there were no differences. These results indicate that love does not influence births, at least in this cultural context.

### Magnitude of parent–offspring conflict

If parents and offspring agree on the ideal traits in a potential in-law/spouse then they should theoretically prefer the same individuals and thus similar reproductive outcomes would be achieved in arranged and non-arranged marriages. Broader evidence indicates that parents and offspring do not agree on the desired traits in a spouse^4–7,10^. However, these prior studies do not guarantee that parent–offspring disagreement exists in every context, and any society with low parent–offspring disagreement might not show the expected fitness differentials. Based on the available descriptive data, there is low parent–offspring disagreement in this population, as has been expressed in focus group discussions in other Nepali contexts^33^. This offers one potential explanation why fitness does not differ between those whose parents choose their spouse and those who choose their own spouse.

However, the limited scope of questions asked in the CVFS may lead to underestimating disagreement in this context. Parents and offspring were only asked about their first and second most-preferred traits and were not asked about undesirable traits. Previous surveys comparing parent and offspring preferences show the highest levels of disagreement over mid-ranking traits^5,39^; thus, only probing the top two most preferred qualities would miss such disagreement. Parents and offspring were also not asked about traits shown to produce substantial disagreement in previous studies, such as similar ethnic or religious background. In Nepal, offspring choosing spouses from outside sanctioned caste or ethnic groups may incite considerable parent–offspring disagreement.

Even if there is unmeasured parent–offspring disagreement in this context, parents and offspring may compromise on their desires, or they may be able to find spouses that meet the desires of both parents and offspring in actual spouse-choice decisions^33^. Even when disagreement does occur, it is possible that parents choose better spouses for their children than children would choose for themselves based on additional ecological knowledge, better assessment of longer-term fitness benefits, and their greater bargaining power in the mating market and over their offspring. These diverse factors could help explain why there are no fitness differences between arranged and non-arranged marriages in these analyses.

### Possible compensatory effects of arranged marriage

It is possible that both arranged and non-arranged marriages are beneficial for fitness but in different ways. While, theoretically, freely choosing a partner could lead to better fitness via genetic compatibility, having a parent choose a mate could also provide fitness benefits via social ties within and across communities^40^. In Nepal and other contexts, it is not uncommon for couples who marry against their parents’ wishes to be ostracized or discriminated against in their communities, making social support a tangible benefit of arranged marriage^4,33^. Parents may also provide direct “compensation” for offspring who accept their parents’ wishes, such as larger inheritances, higher marital payments, or additional grandparental care. Thus, if couples in arranged marriages receive more financial and/or social benefits in ways not identified in the current study, such compensation may offset other costs of arranged marriage, thereby contributing to the null results in this study. These benefits of arranged marriage may be especially important for long-term or intergenerational buffering against environmental, social, or epidemiological crises. This possibility should be considered in future research, but such data were unavailable from the CVFS database at the time of analysis.

### Fitness measures

In countries like Nepal, where the demographic transition is well underway, variation in measures of fertility and offspring survival may be limited. Furthermore, this area of Nepal has ample access to prenatal and perinatal care, so offspring survival may be equalized by healthcare access. However, given the consistency between the results of this present analysis and previous analyses of fitness and spouse choice^30,41^ this result is likely not simply a result of the Nepali context. It is also possible that differences in fertility outcomes are small and the analyses presented here have low statistical power to detect differences between groups. Measures of offspring health or growth were not available at the time of these analyses, but these outcomes may differ based on marriage type if self-chosen mates confer “good genes” better than parentally-chosen mates. Thus, examination of a wider range of fitness outcomes may provide more information about the different benefits offered by parental and offspring mate choice.

## Conclusions

Women whose parents chose their spouse, women who chose their own spouse, and women who chose a spouse in collaboration with their parents show no differences in total births, offspring survival, or interbirth intervals to their first and to their second child. This is surprising because individual mate preferences likely evolved to target fitness-relevant traits, and thus individuals who exercise their own mate preferences by choosing a spouse should have higher fitness. The failure of arranged marriage to produce these predicted effects has now been replicated across multiple populations with different cultural and ecological contexts^30,41^. There are several potential reasons for this set of findings. First, the most fitness-relevant elements of spouse choice may not be captured by dichotomous or trichotomous marriage types. Assessing spouse choice from a series of questions about who initially found the spouse, whether parents and offspring communicated about a potential spouse, and whether either party gave approval can produce more precision on the degree of independence in spouse choice. Second, parent–offspring disagreement over preferred traits in a spouse appears to be low in this community. If there is minimal disagreement in this or other cultures with arranged marriages, then fitness differences would not be expected between those choosing their own spouse and those whose parents are choosing their spouse. Lastly, involving parents in marriage decisions may provide other benefits that could compensate for any fitness costs of not independently choosing a mate. Large marital transactions, inheritances, trade networks, alliances, and/or additional grandparental care may even outweigh the benefits of choosing a mate independently. If this is the case, independent mate choice and parental mate choice may have similar fitness consequences, making human mate choice unique among animals.

## Methods

### Data

The CVFS is directed and maintained by Axinn et al.^34^ at the University of Michigan. The data in this analysis were collected via individual interviews in 2008, in which all men and women aged 15–34 and their parents in 151 sample neighborhoods were surveyed. The sample neighborhoods represent a spread of rural and urban communities (ranging 0.02–17.7 miles from the nearest city, mean = 8.24, SD = 3.93). The data set also includes migrants who have moved away from sample neighborhoods for work, but who still have spouses or children in the sample neighborhoods. Thus, the data are highly representative of the sampled communities. In Nepal, during the period these data were collected, the total fertility rate was 2.7 and the infant mortality rate was 40 per 1000 births^42^. The following analyses limited this sample to women who were ever married and who completed both the individual interviews and the life history calendar (n = 1544). The CVFS data are available upon request via the Inter-University Consortium for Political and Social Research (ICPSR). Because this work uses secondary analyses of existing data, it was exempted from human subjects review by the University of California, Santa Barbara’s Institutional Review Board (Protocol 38-16-052). The Chitwan Valley Family Study is carried out in accordance with relevant guidelines and regulations, and it is approved by the University of Michigan’s Institutional Review Borad (FWA 00,004,969) and the Nepal Health Research Council. Informed consent was obtained from all subjects and/or their legal guardians.

#### Marriage type

Responses to the question “Who primarily was responsible for choosing your spouse?” were used to determine marriage type for each respondent. Respondents were prompted to choose “Parents/other relatives”, “Myself”, or “Both” as responses. If they answered “Both”, they were asked again to choose whether their parents or themselves had more influence. Marriage type was categorized as non-arranged if the respondent answered “Myself” in either the first or second round of questioning. Marriage type was categorized as arranged if the respondent answered “Parents/other relatives” in the first or second round of questioning. Respondents who maintained that both themselves and their parents were equally responsible for choosing their spouse following the second round of this question (n = 49) were excluded from the analyses using dichotomous spouse choice. To determine whether our results were simply a result of dichotomous categorization, which does not accurately capture the range of ethnographic descriptions of spouse choice methods, analyses were also run using three categories of marriage types based on the respondents’ first responses, meaning those who responded “Both” were kept in a separate category, hereafter called co-selected marriages (see Fig. 1).

#### Fitness variables

Many variables were derived from life history calendars incorporating community and national events to improve recall among respondents who do not know exact dates or ages^43^. Total number of births, number and timing of offspring deaths, and the timing of each birth were derived from life history calendars of the respondents. Life history calendars were also used to calculate each respondent’s age, birth year, and age at first marriage, which were used as controls in several models. The total years on birth control were also calculated from life history calendars. However, I do not include birth control in the models reported in this paper because contraceptive use is a method through which women in different marriage types could produce the differences in fitness outcomes that we are interested in.

#### Socioeconomic variables

Because arranged marriages may be associated with certain demographic characteristics that could confound any effect of marriage type, like income and education, I controlled for additional socioeconomic factors. Education was determined by the respondent’s self-reported highest level of education completed. This was either listed as the highest grade level completed, or the highest degree obtained. Degrees obtained were converted to a year measure based on the typical number of years it takes to complete each degree type (e.g. a School Leaving Certificate would be 10 years, A-levels would be 12 years, and a bachelor’s degree would be 16 years). Annual household income level contains seven categories specified in Nepalese Rupees (< 10,000 [under $100], 10–25,000, 25–50,000, 50–100,000, 100–250,000, 250–500,000, and > 500,000 [above $4000]). Interviewers began by asking whether household income was higher or lower than 50,000 Rupees (about $400 USD), and then asking the same for each subsequent category until the range of the household income was determined. Income data was not collected for individuals and was not available for every household in the data set. Individuals without household income data, who were distributed across marriage types but tended to be younger with fewer births, were excluded from all analyses (n = 246).

### Analysis

All data analysis was done using R^44^. Variance Inflation Factors for each model were calculated to check for collinearity (the max VIF in any model was 1.28).

For Analysis Set 1, I examined the relationship between marriage type and the count of total births using Poisson generalized linear models. Analysis 1a predicts total number of births for women as a function of age, household income, education, age at first marriage, and dichotomous marriage type to allow for comparison to previous studies^30,41^. Model 1b expands 1a by using the same controls with the trichotomous grouping of marriage types.

Analysis Set 2 examines the relationship between marriage type and offspring survival to age 15 (i.e., 0 to 15 years following a birth event). Because the CVFS does not collect exact dates of life events, these analyses used discrete time survival analysis^37^, which uses a logistic regression to predict whether an event occurred in each one-year period. Because these analyses (unlike other types of survival analyses) provide separate estimates for time and marriage type, model estimates are used to predict hazard ratios and survival curves (Fig. 3) to examine how the effect of marriage type varies over time. All models include covariates for time since birth, mother’s age at the time of the birth, child’s birth year, mother’s education, and household income level. Similar to Analysis Set 1, I ran a model with dichotomous spouse choice (2a) that included data on 4329 births from 1206 women and one with trichotomous spouse choice (2b) that included data on 4454 births from 1250 women.

In Analysis Set 3, I examined whether marriage type affected the rate at which individuals have children. To examine first-birth interval (Analysis 3a) and second-birth interval (Analysis 3b), I again used discrete-time survival analysis^37^. For these analyses, our period of observation was the first nine years following their marriage (first-birth interval) or nine years following the first birth (second-birth interval), at which time 95% of women had experienced a birth event. Time and time squared were included in all analyses to better represent the non-linearity in the raw data, and controls for age, age at marriage, education, and household income were included. The first- and second-birth-interval models were also run with trichotomous marriage type (Models 3c and 3d). Women who never experienced a first birth (78 of the 1249 women in the dichotomous analyses and 85 of the 1298 women in the trichotomous analyses) were not included in models examining time to second birth.

### Supplementary Information

Supplementary Information.

## Data Availability

This research uses data from the CVFS, whose PI is William G. Axinn. The CVFS is a longitudinal study and data collection is funded by multiple grants [No. RO1-HD032912, RO1-HD033551 and 5 R37 HD039425) from the National Institute of Health, Eunice Kennedy Shriver National Institute of Child Health and Human Development and Fogarty International Center. Persons interested in obtaining data files from the CVFS should contact the Inter-University Consortium for Political and Social Research, The University of Michigan, Institute for Social Research, P.O. Box 1248, Ann Arbor, MI 48,106–1248, netmail@icpsr.umich.edu.
