# Supplementary material for: Arranged and non-arranged marriages have similar reproductive outcomes in Nepal
Source: Sci Rep. 2024 May 14;14:11080. doi: 10.1038/s41598-024-61467-8 (PMC11094175; doi:10.1038/s41598-024-61467-8)

**Supplementary Information**

Title: Arranged and non-arranged marriages have similar reproductive outcomes in Nepal

Author: Elizabeth Agey

Supplementary Table S1: Descriptive statistics for dichotomous spouse choice categories for women in the Chitwan Valley Family Study (CVFS) Sample

|  |  | Non-arranged (n=778) | | Arranged (n=717) | |
| --- | --- | --- | --- | --- | --- |
| Statistic | | Mean | St. Dev. | Mean | St. Dev. |
| Age*** | | 38.0 | 10.6 | 45.3 | 10.4 |
| Age at Marriage*** | | 21.9 | 4.0 | 20.7 | 4.7 |
| Marriage Year*** | | 1993 | 10.9 | 1984 | 12.4 |
| Age at First Birth | | 24.1 | 4.2 | 24.2 | 5.0 |
| Total Children*** | | 2.8 | 2.0 | 3.9 | 2.3 |
| Total Surviving Children*** | | 2.6 | 1.7 | 3.5 | 1.9 |
| Total Years on Birth Control** | | 3.3 | 4.2 | 2.7 | 4.2 |
| Years Attended School | | 6.1 | 4.4 | 5.8 | 4.5 |
| Household Income Level | | 3.2 | 1.3 | 3.3 | 1.4 |
|  | *** indicates means are significantly different at p<.001  ** indicates means are significantly different at p<.05 | | | | |

Supplementary Table S2: Responses to the question “How much do you love your spouse?” for the married women included in subsequent analyses.

| Marriage Type | Very Much | Some | A little or Not at all | Total |
| --- | --- | --- | --- | --- |
| Arranged | 137 (25%) | 312 (57%) | 98 (18%) | 547 |
| Self-Selected | 183 (30%) | 331 (54%) | 103 (17%) | 617 |
| Co-Selected | 100 (29%) | 193 (56%) | 53 (15%) | 346 |

Overall differences: χ^2^=4.21, p=.65

Supplementary Table S3: Responses to the question “How frequently do you and your spouse disagree?” for the married women included in subsequent analyses.

|  | Frequently or Sometimes | Seldom | Never | Total |
| --- | --- | --- | --- | --- |
| Arranged | 67 (12%) | 331 (61%) | 148 (27%) | 546 |
| Self-Selected | 110 (18%) | 368 (60%) | 140 (23%) | 618 |
| Co-Selected | 40 (12%) | 211 (61%) | 95 (27%) | 346 |

Overall differences: χ^2^=14.92, p=.02

Supplementary Table S4: Results of models 1a and 1b. Coefficients are converted to rate ratios and standard errors are in parentheses. Reference spouse choice group for all models is arranged marriage (parentally selected).

|  | | |
| --- | --- | --- |
|  | *Dependent variable:* | |
|  |  | |
|  | Total Children | |
|  | Model 1a | Model 1b |
|  | | |
| Self-Selected Spouse | 0.996 | 1.026 |
|  | (1.034) | (1.038) |
|  |  |  |
| Co-Selected Spouse |  | 0.964 |
|  |  | (1.046) |
|  |  |  |
| Age | 1.037^***^ | 1.037^***^ |
|  | (1.002) | (1.002) |
|  |  |  |
| Household Income Level | 1.000 | 1.002 |
|  | (1.012) | (1.012) |
|  |  |  |
| Years in School | 0.982^***^ | 0.984^***^ |
|  | (1.004) | (1.004) |
|  |  |  |
| Age at Marriage | 0.969^***^ | 0.968^***^ |
|  | (1.004) | (1.004) |
|  |  |  |
| Intercept | 1.440^***^ | 1.434^***^ |
|  | (1.120) | (1.123) |
|  |  |  |
|  | | |
| Observations | 1,249 | 1,298 |
| Log Likelihood | -2,210.443 | -2,294.311 |
| Akaike Inf. Crit. | 4,432.885 | 4,602.621 |
|  | | |
| *Note:* | ^***^p<0.01 | |

Table S5: Table of model results for Models 2a-b, survival to age 15. Coefficients are converted to rate ratios and standard errors are in parentheses. Reference spouse choice group is arranged marriage.

|  | Model 2a:  Dichotomous Choice | Model 2b:  Trichotomous Choice |
| --- | --- | --- |
| Time Since Birth | 0.58 ***  (1.03) | 0.58 ***  (1.03) |
| Self-Selected Spouse | 1.01  (1.12) | 1.12  (1.13) |
| Co-Selected Spouse | - | 0.97  (1.19) |
| Years in School | 0.93 ***  (1.01) | 0.94 ***  (1.01) |
| Household Income Level | 1.00  (1.04) | 1.01  (1.04) |
| Mother’s Age at Birth | 0.98  (1.01) | 0.99  (1.01) |
| Child’s Birth Year | 0.94 ***  (1.01) | 0.93 ***  (1.01) |
| Intercept | 130.28 (14.11) *** | 135.99 (14.22) *** |
| N^a^ | 61,775 | 63,685 |
| AIC | 3390.7 | 3443.6 |

***p<.001

a. N is each year (0-15) following each birth event for each respondent. Model 2a includes 4,329 birth events from 1,206 women. Model 2b includes 4,454 birth events from 1,250 women.

Supplementary Table S6: Table of model results for Models 3a-3d. Coefficients are converted to rate ratios and standard errors are in parentheses. Reference spouse choice group is non-arranged marriage (for dichotomous choice) or self-selected marriage (for trichotomous choice).

|  | Dichotomous Choice | | Trichotomous Choice | |
| --- | --- | --- | --- | --- |
|  | Model 3a: First Birth Interval | Model 3b: Second Birth Interval | Model 3c: First Birth Interval | Model 3d: Second Birth Interval |
| Time to Birth Event | 2.84 ***  (1.058) | 2.646 ***  (1.069) | 2.91 ***  (1.057) | 2.66 ***  (1.068) |
| Time Squared | 0.89 ***  (1.008) | 0.854 ***  (1.012) | 0.89 ***  (1.008) | 0.85 ***  (1.012) |
| Arranged Marriage | 0.82 **  (1.083) | 1.017 ***  (1.087) | 0.73 ***  (1.093) | 0.94  (1.098) |
| Co-Selected | - | - | 0.92  (1.102) | 1.17  (1.109) |
| Respondent’s Age | 0.97 ***  (1.004) | 1.022  (1.022) | 0.97 ***  (1.004) | 1.02 ***  (1.004) |
| Respondent’s Age at Marriage | 1.07 ***  (1.008) | 0.996  (1.009) | 1.07 ***  (1.008) | 0.99  (1.009) |
| Years in School | 1.03 ***  (1.029) | 0.996  (1.010) | 1.03 ***  (1.009) | 0.99  (1.009) |
| Household Income Level | 1.04  (1.029) | 1.035  (1.030) | 1.04  (1.028) | 1.04  (1.029) |
| Intercept | 0.099 ***  (1.291) | 0.082 ***  (1.329) | 0.099 ***  (1.286) | 0.08 ***  (1.322) |
| N^a^ | 4,708 (1,249) | 3,497 (1,170) | 4,854 (1,298) | 3,603 (1,213) |
| Log Likelihood | -2319.41 | -1978.758 | -2391.24 | -2042.37 |
| AIC | 4654.81 | 3973.516 | 4800.485 | 4102.74 |

***p<.001, **p<.01

a. N in these models represents the number of respondents multiplied by each unit of time. The parentheses note the total number of unique respondents represented in each model.

Supplementary Table S7: Most preferred trait in a spouse or in-law for children and their parents, respectively, for 3,307 parent-offspring dyads in the CVFS. Shaded squares represent concordance in parent and offspring choices.

|  | Parents | | | | |
| --- | --- | --- | --- | --- | --- |
| Children | Physical Beauty | Education | High Paying Job | Someone he/she loves | Row Totals |
| Physical Beauty | 33 (1%) | 132 (4%) | 62 (2%) | 19 (<1%) | 246 (7%) |
| Education | 180 (5%) | 1,034 (31%) | 479 (14%) | 142 (4%) | 1,835 (55%) |
| High Paying Job | 78 (2%) | 447 (14%) | 354 (11%) | 54 (2%) | 933 (28%) |
| Someone You Love | 38 (1%) | 152 (5%) | 73 (2%) | 30 (1%) | 293 (9%) |
| Column Totals | 329 (10%) | 1,765 (53%) | 968 (29%) | 245 (7%) | 3,307 |

Supplemental Figure S1: The gap between parent and offspring responses to the question “How soon do you want your child to marry [for parents]/want to marry [for offspring]”. 0 indicates that parents and offspring chose the same interval (e.g., both said “in 5 years”), while numbers represent the number of years between their responses. Negative numbers indicate that offspring want to marry sooner than their parents desire. Positive numbers indicate parents want their offspring to marry sooner than the offspring desired.


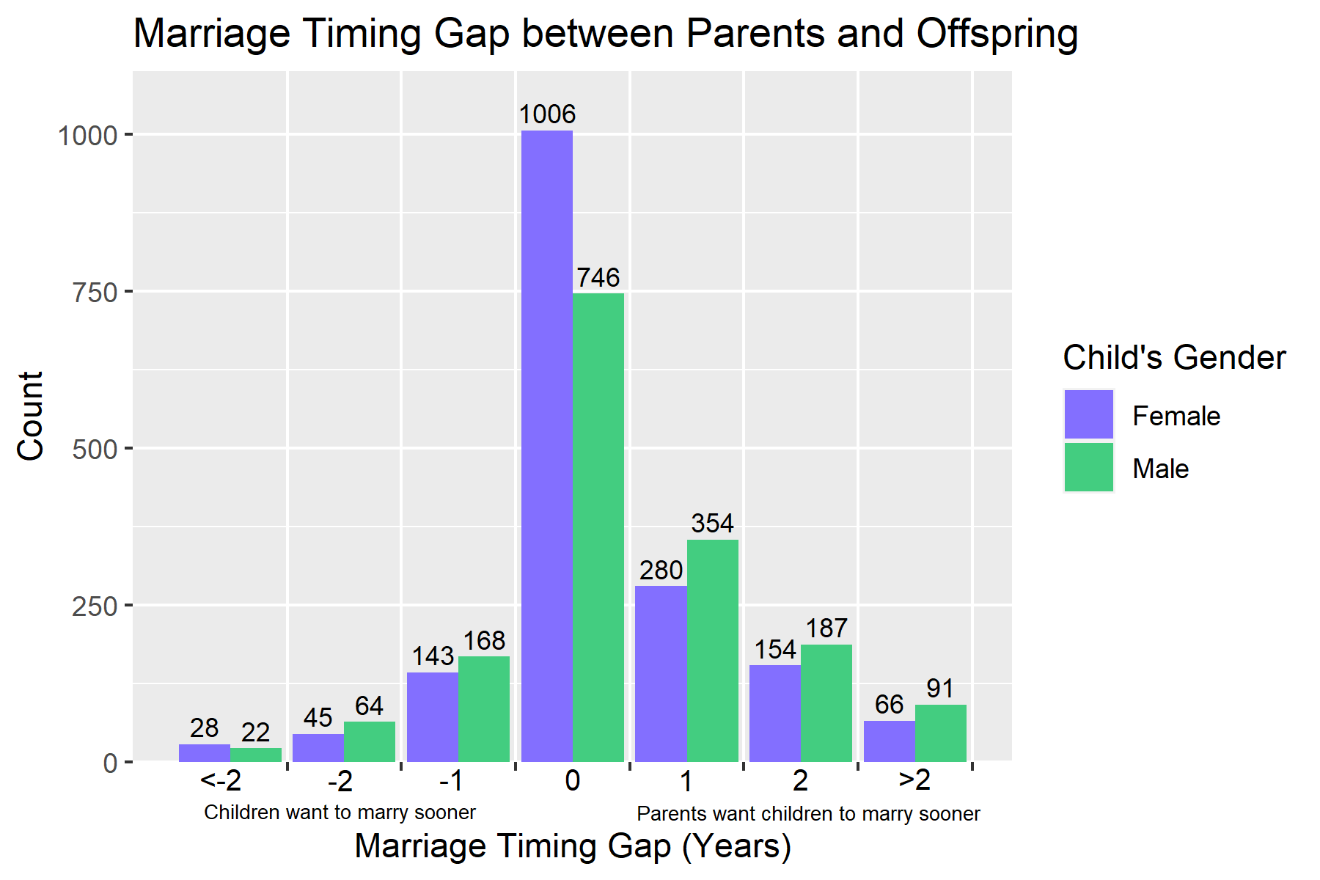

Supplement: Supplementary file 1 — Supplementary Information. [file 41598_2024_61467_MOESM1_ESM.docx]
